# Assessing for race, ethnicity, and socioeconomic disparities in central line-associated bloodstream infection risk in a large academic health system

**DOI:** 10.1017/ice.2024.133

**Published:** 2024-12

**Authors:** Lindsey B. Gottlieb, Radhika Prakash-Asrani, William Dube, Zanthia Wiley, Giancarlo Licitra, Scott K. Fridkin

**Affiliations:** 1 Division of Infectious Diseases, Department of Medicine, School of Medicine, Emory University, Atlanta, GA, USA; 2 Department of Epidemiology, Rollins School of Public Health, Emory University, Atlanta, GA, USA; 3 Division of General Internal Medicine, Department of Medicine, School of Medicine, Emory University, Atlanta, GA, USA

## Abstract

**Objective:**

To examine the relationship between race and ethnicity and central line-associated bloodstream infections (CLABSI) while accounting for inherent differences in CLABSI risk related to central venous catheter (CVC) type.

**Design:**

Retrospective cohort analysis.

**Setting:**

Acute care facilities within an academic healthcare system.

**Patients:**

Adult inpatients from January 2012 through December 2017 with CVC present for ≥2 contiguous days.

**Methods:**

We describe variability in demographics, comorbidities, CVC type/configuration, and CLABSI rate by patient’s race and ethnicity. We estimated the unadjusted risk of CLABSI for each demographic and clinical characteristic and then modelled the effect of race on time to CLABSI, adjusting for total parenteral nutrition use and CVC type. We also performed exploratory analysis replacing race and ethnicity with social vulnerability index (SVI) metrics.

**Results:**

32,925 patients with 57,642 CVC episodes met inclusion criteria, most of which (51,348, 89%) were among non-Hispanic White or non-Hispanic Black patients. CVC types differed between race/ethnicity groups. However, after adjusting for CVC type, configuration, and indication in an adjusted cox regression, the risk of CLABSI among non-Hispanic Black patients did not significantly differ from non-Hispanic White patients (adjusted hazard ratio [aHR] 1.19; 95% confidence interval [CI]: 0.94, 1.51). The odds of having a CLABSI among the most vulnerable SVI subset compared to the less vulnerable was no different (odds ratio [OR] 0.95; 95% CI: 0.75–1.2).

**Conclusions:**

We did not find a difference in CLABSI risk between non-Hispanic White and non-Hispanic Black patients when adjusting for CLABSI risk inherent in type and configuration of CVC.

## Introduction

Racial disparities amongst health outcomes have been described as far back as the 1970s.^1^ More recently, racial disparities have been identified in the quality of healthcare patients receive, influencing patient safety outcomes.^2^ However, available literature is conflicting as to whether such disparities exist specifically amongst healthcare-associated infections (HAIs).^3–6^ Socioeconomic factors such as income, language barriers, and access to transportation may contribute to racial disparities in risk for HAIs.^7^ Discerning the relative contribution to risk for HAIs attributed to race and ethnicity, or socioeconomic factors linked to race and ethnicity, while accounting for underlying illness, healthcare delivery setting, and major HAI risk factors has not been evaluated well. The aim of this study is to utilize data from a previously studied cohort of patients in a large academic health system to further examine the relationship between race, ethnicity, and social vulnerability with central line-associated bloodstream infections (CLABSI) while accounting for inherent differences in risk related to device utilization.^8^

## Methods

### Study population, data source and design

We performed a retrospective analysis of a cohort of inpatients admitted from January 1, 2012 to December 31, 2017 to four acute care hospitals in Emory Healthcare (EHC, Atlanta, GA, USA). These included Hospital A (suburban, non-profit, 582 beds, 46.1% are non-Hispanic [NH] Black), Hospital B (Urban, non-profit, 537 beds, 71.6% are NH Black), Hospital C (Suburban, non-profit, 373 beds, 32.4% are NH Black), and Hospital D (suburban, non-profit, 152 beds, 19.7% are NH Black). Patients eligible for inclusion were adult (≥18 years age) inpatients with central venous catheters (CVC) present for at least two contiguous days, with a length of stay ≤ 50 days (the 95^th^ percentile of length of stay), with ≤ 3 concurrent CVCs (defined as the presence of at least two CVCs on at least two consecutive days), and with < 6 unique CVC episodes during their hospitalization. These cutoffs were chosen to eliminate outliers and have results reflect the more typical CVC experience. Patients were included regardless of whether their CVC was present on admission or placed during their hospitalization.

Encounter data was extracted from the EHC clinical data warehouse and included demographics (age, gender, race and ethnicity), International Classification of Diseases, Ninth Edition, Clinical Modification (ICD-9-CM) discharge codes (allowing calculation of Charlson Comorbidity Index [CCI]), and orders for total parenteral nutrition (TPN) and chemotherapy. Race and ethnicity were assigned to mutually exclusive groups of Hispanic, NH Black, NH White, Other NH, and Unknown based on data entered into the medical record via facility-specific intake procedures, which were not standardized during the study period and thus reflect a mix of patient-report and assignment by the registrar. We obtained central venous catheter (CVC) insertion/removal data from a dedicated CVC tracking system maintained and validated by our research team. CLABSI data was extracted from EHC surveillance data reported to the National Healthcare Safety Network (NHSN), which excluded infections categorized as mucosal barrier injury, and linked to patient encounters.^9^

The impact of socioeconomic factors was assessed utilizing several metrics derived from CDC’s Social Vulnerability Index (SVI). The SVI includes measures of social and economic segregation such as household composition, housing, transportation, race and ethnicity, and language and can be considered a better marker of social disparities than race and ethnicity.^10^ For this analysis, the home address of each patient was geocoded using ARC GIS (Version 10.8.2, ESRI) and assigned values for each theme of the SVI. SVI data was accessed from the CDC/ATSDR SVI Data and Documentation Download database.^10^ Georgia census tract-level data from 2020 was used.

### Designation of CVC episodes

The type of CVC was considered the key exposure variable and was mapped into two mutually exclusive categories based on CLABSI risks identified from our previous study: lower-risk (ports, peripherally inserted central venous catheters [PICCs], hemodialysis CVCs) or higher-risk (temporary CVCs, e.g., short-term tunneled/non-tunneled, introducers, pulmonary artery catheters).^8^ Hemodialysis CVCs included both non-tunneled and tunneled CVCs recorded as dedicated for dialysis/pheresis. Dwell times (removal date-insertion date) and periods of overlapping dates (i.e. concurrence) were determined. For those patients who developed a CLABSI, central line days were only counted to the date of CLABSI.

Each encounter was divided into distinct CVC episodes that included either a single CVC per episode (either serial CVCs or a single CVC) or concurrent CVCs being used on overlapping dates. Patients could contribute to both single CVC episodes (limited to dates with single CVC) and concurrent CVC episodes (limited to dates with concurrent CVCs). For any single patient encounter, a CLABSI was attributed to the CVC episode that included the CLABSI date or was within two days prior to the CLASBSI date, if the CLABSI date was after CVC removal.

### Statistical approach

Initial descriptive analysis included all patient encounters and CVC episodes up to the time of first CLABSI. For patients with multiple CVC episodes, patients’ underlying illnesses and demographics were extracted from the encounter corresponding to the highest risk CVC episode. Using logistic regressions, we estimated the unadjusted risk of CLABSI for each demographic and clinical characteristic including CVC type and use of TPN or chemotherapy. Continuous variables were assessed by Wilcoxon rank-sum test. Cox regression with a Heaviside function at 14 days was used to model the effect of race on time to CLABSI, adjusted for TPN use and CVC type. Based on previous analysis of these data, we categorized CVC episodes into 3 distinct categories based on risk for CLABSI: single lower-risk CVC episode, single higher-risk CVC episode, and concurrent CVC episode (concurrent CVC use of any types).^8^ The Heaviside function was used at 14 days because the hazard ratio was essentially 1.0 with survival curves indistinguishable from each other until day 14 when the curves began to separate. Healthcare system-wide and facility-specific Kaplan-Meier disease-free survival curves were generated. We limited the survival analysis to the first 21 days of hospitalization to minimize the impact and bias of extremely long inpatient stays. Adjusted hazard ratios (aHR) and 95% confidence intervals (CIs) were calculated using SAS software (version 9.4, Cary, NC).

Because of the lack of consistent findings evaluating the association of race and ethnicity with CLABSI, we performed exploratory analysis replacing race with SVI metrics.^11^ Each of the variables of the SVI were ranked across all census tracts in Georgia and then assigned a corresponding percentile rank (percentile rank = (rank–1)/(n–1)). The sum of the percentile ranks of each variable within each domain were used in this analysis to create a theme percentile rank, as well as a composite theme that included the overall rank for the census tract that incorporates all themes.^10^ Notably, higher percentile in any SVI theme correlates with more social disadvantage. The primary analysis compared CLABSI risk among patients most vulnerable (highest quartile rankings) to less vulnerable (other quartile rankings) by univariate logistic regression. In addition, we evaluated the difference in distribution of the SVI metrics between patients with CLABSI and those without through univariate analysis by Wilcoxon rank sum test.

This study was reviewed and approved by the Emory IRB by expedited process under 45 CFR.46.110 and/or 21 CFR 56.110 because it poses minimal risk and fits expedited review category F[5] as set forth in the Federal Register.

## Results

Overall, 32,925 patients had at least one hospital admission with at least 2 days of CVC use resulting in 57,642 CVC episodes. At the time of the highest risk CVC episode, patients were most likely to be hospitalized at Hospital A (51%), followed by Hospital B (27%), then Hospital C (17%) and D (4%). CVC use for hemodialysis (14%) or TPN (11%) was relatively common, while use for chemotherapy was rare (2%). Most patients were NH White or NH Black (49% and 39% respectively) with the remaining Hispanic (2%), Other NH race (3%) or Unknown race (7%) (Table 1).

**Table 1.** Demographics and clinical characteristics of patients with CVCs, stratified by race and ethnicity

Characteristic	Total (N = 32,925)	NH Black (N = 12,737)	NH White (N = 16,163)	Hispanic (N = 695)	Other NH (N = 921)	Unknown (N = 2,409)
Female Sex, N (%)	16,411 (50%)	7,173 (56%)	7,406 (46%)	3,118 (46%)	420 (46%)	1,094 (45%)
Age, years, median (IQR)	59 (47–68)	58 (46–68)	64 (53–72)	56 (43–67)	60 (48–70)	54 (42–64)
Underlying Illness, N (%)						
CHF	7,365 (23%)	3,373 (27%)	3,403 (21%)	139 (20%)	181 (20%)	539 (22%)
PVD	4,729 (14%)	1,703 (13%)	2,508 (16%)	85 (12%)	105 (11%)	328 (14%)
DM (with complications)	4,626 (14%)	2,455 (19%)	1,713 (11%)	92 (13%)	129 (14%)	237 (10%)
CKD	11,006 (33%)	5,728 (45%)	4,185 (26%)	190 (27%)	257 (28%)	592 (25%)
Malignancy	10,927 (33%)	3,988 (31%)	5,773 (36%)	266 (38%)	353 (38%)	547 (23%)
CCI Score, median (IQR)	4 (2–7)	5 (2–8)	4 (2–6)	4 (2–7)	4 (2–7)	3 (2–6)
Receiving chemotherapy, N (%)	595 (2%)	208 (2%)	317 (2%)	21 (3%)	21 (2%)	28 (1%)
Receiving TPN, N (%)	3,661 (11%)	1,214 (10%)	2,054 (13%)	58 (9%)	97 (11%)	238 (10%)
Receiving hemodialysis, N (%)	4,634 (14%)	2,734 (22%)	1,383 (7%)	103 (15%)	129 (14%)	285 (12%)
CVC Risk Category, N (%)						
1 (Single lower risk CVC)^a^	16,218 (49%)	6,663 (52%)	7,694 (48%)	349 (50%)	474 (52%)	1038 (43%)
2 (Single higher risk CVC)^a^	10,247 (31%)	3,351 (26%)	5,428 (34%)	214 (31%)	302 (33%)	898 (37%)
3 (Concurrent CVC)^b^	6,460 (28%)	2,723 (21%)	2,987 (19%)	132 (19%)	145 (16%)	473 (20%)
CVC Dwell Time, days, median (IQR)	5 (3–9)	5 (3–9)	5 (3–8)	5 (2–10)	5 (3–9)	5 (3–10)
Length of Stay, days, Median (IQR)	10 (6–17)	10 (6–17)	10 (6–16)	11 (6–17)	10 (6–17)	12 (7–18)
CLABSIs, N (%)	526 (1.6%)	217 (1.7%)	256 (1.6%)	11 (1.6%)	8 (0.9%)	34 (1.4%)

Note. CVC, central venous catheter; NH, non-Hispanic; IQR, interquartile range; CHF, congestive heart failure; PVD, peripheral vascular disease; DM, diabetes mellitus; CKD, chronic kidney disease; CCI, Charlson Comorbidity Index; TPN, total parenteral nutrition; CLABSI, central line-associated bloodstream infection.

a Lower-risk includes ports, PICCs [peripherally inserted central catheter], and hemodialysis CVCs; higher risk includes temporary CVC types (e.g., short-term tunneled/non-tunneled, introducers, pulmonary artery catheters)

b Concurrence was defined as any 2 CVCs present for 2 or more of the same days

Concurrent CVC use occurred at least once in 6,420 (20%) patients. During these concurrent CVC episodes, CVC use was relatively common for hemodialysis (1926, 30%) and TPN (1,057, 16%) (Table 2). Use of only single lower-risk CVCs was most common, occurring in 16,218 patients (49%), while use of at least one single higher-risk CVC occurred in 10,247 (31%) patients. Compared to patients with only use of single lower-risk CVCs, patients with an episode of single higher-risk CVC use were less likely to be NH Black compared to NH White and 40% more likely to develop a CLABSI (Table 2). Patients with at least one episode of concurrent CVC use were of equal likelihood to be NH Black or NH White, more likely to be receiving TPN or hemodialysis, and three-fold more likely to develop a CLABSI, when compared to patients with only use of single lower-risk CVCs (Table 2).

**Table 2.** Likelihood of exposure to highest risk CVCs, by race/ethnicity and clinical characteristics using unadjusted multinomial logistic regression

Characteristic	Risk category of patient’s highest risk CVC episode, N (%)			Odds ratio (95% CI)	
	1 – Single lower-risk^a^, 16,218	2 – Single higher-risk^a^, 10,247	3 – Concurrent^b^, 6,460	Single higher-risk^a^ vs. single lower-risk^a^	Concurrent^b^ vs. single lower-risk^a^
Race/Ethnicity^c^					
NH Black	6,663 (41%)	3,351 (33%)	2,723 (42%)	0.71 (0.67, 0.75)	1.05 (0.99, 1.12)
NH White	7,694 (47%)	5,482 (53%)	2,987 (46%)	Ref	Ref
Hispanic	349 (2%)	214 (2%)	132 (2%)	0.86 (0.72, 1.02)	0.97 (0.79, 1.20)
Other NH	474 (3%)	302 (3%)	145 (2%)	0.89 (0.77, 1.04)	0.79 (0.65, 0.95)
Receiving chemotherapy	441 (3%)	46 (<1%)	108 (2%)	0.16 (0.12, 0.22)	0.61 (0.49, 0.75)
Receiving TPN	2,037 (13%)	567 (6%)	1,057 (16%)	0.41 (0.37, 0.45)	1.36 (1.26, 1.48)
Receiving hemodialysis	2,708 (17%)	0 (0%)	1,926 (30%)	NA	2.12 (1.98, 2.27)
CLABSI	92 (1%)	82 (1%)	122 (2%)	1.41 (1.05, 1.91)	3.37 (2.57, 4.43)

Note. CVC, central venous catheter; CI, confidence interval; NH, non-Hispanic; TPN, total parenteral nutrition; CLABSI, central line-associated bloodstream infection.

a Lower-risk includes ports, PICCs [peripherally inserted central catheter], and hemodialysis CVCs; higher-risk includes temporary CVC types (e.g., short-term tunneled/non-tunneled, introducers, pulmonary artery catheters)

b Concurrence was defined as any 2 CVCs present for 2 or more of the same days

c Unknown race = 2409, 7% of all data

Most of the 57,642 CVC episodes (51,348, 89%) were among NH White or NH Black patients. Limiting further analysis to only NH White and NH Black patients, the majority contributed a single CVC episode (65%), with fewer contributing 2 (20%), 3 (7%), 4 (3%) or more (5%) episodes. These episodes were characterized by similar dwell times for single CVC episodes (median 5 days, interquartile range [IQR] 3–10 days) and concurrent CVC episodes (median 6 days, IQR 3–11 days). In this subset, CVC episodes ending in a CLABSI had significantly longer dwell times (median 13 days, IQR 7–20 days) than those without a CLABSI (median 5 days, IQR 3–8, *P* < 0.001) (Table 3). Odds of having a CLABSI were significantly greater among CVC episodes used for TPN or chemotherapy and significantly greater among single higher-risk CVC or concurrent CVC episodes when compared to single lower-risk CVC episodes. CVC episodes among NH Black patients had no excess risk for CLABSI compared to those among NH White patients (Table 3).

**Table 3.** Risk of CLABSI by patient demographics and clinical characteristics

Characteristic	Total (N = 51,348)	With CLABSI (N = 472)	Without CLABSI (N = 50,876)	Odds ratio (95% CI)^a^
Race, N (%)				
NH Black	24,700 (48%)	216 (46%)	24,484 (48%)	0.9 (0.8–1.1)
NH White	26,648 (52%)	256 (54%)	26,392 (52%)	REF
Age, years, median (IQR)	59 (46–69)	60 (46–67)	59 (46–69)	NA
CCI, median (IQR)	4 (2–7)	4 (2–7)	4 (2–7)	NA
CVC Risk Category, N (%)				
Single lower risk^b^	27,977 (54%)	189 (40%)	27,788 (55%)	REF
Single higher risk^b^	10,359 (20%)	90 (19%)	10,269 (20%)	1.3 (1.0–1.7)
Concurrent CVC^c^	13,012 (25%)	193 (41%)	12,819 (25%)	2.2 (1.8–2.7)
CVC Dwell Time, days median (IQR)	5 (3–8)	13 (7–20)	5 (3–8)	NA
Receiving chemotherapy, N (%)	1,639 (3%)	21 (4%)	1,618 (3%)	1.4 (1.0–2.0)
Receiving TPN, N (%)	5,829 (11%)	106 (22%)	5,723 (11%)	2.3 (1.9–2.7)
Receiving hemodialysis, N (%)	8,924 (17%)	76 (16%)	8,848 (17%)	0.91 (0.71–1.2)

Note. CLABSI, central line-associated bloodstream infection; CI, confidence interval; NH, non-Hispanic; IQR, interquartile range; CCI, Charlson Comorbidity Index; CVC, central venous catheter; TPN, total parenteral nutrition.

a Logistic regressions estimated the unadjusted risk of CLABSI; continuous variables were assessed by Wilcoxon rank-sum test

b Lower risk includes ports, PICCs [peripherally inserted central catheter], and hemodialysis CVCs; higher-risk includes temporary CVC types (e.g., short-term tunneled/non-tunneled, introducers, pulmonary artery catheters)

c Concurrence was defined as any 2 CVCs present for 2 or more of the same days

**Figure 1.**
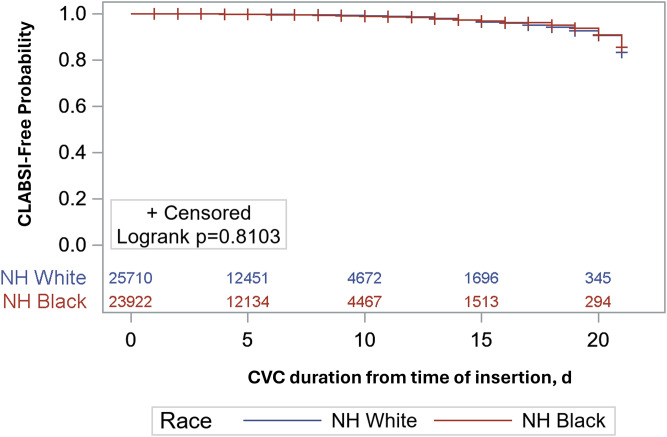
Likelihood of remaining free of CLABSI from day of catheter placement, stratified by Non-Hispanic Black and Non-Hispanic White Race. *Note*: CLABSI, central line-associated bloodstream infection; NH White, non-Hispanic White; NH Black, non-Hispanic Black; d, days

Kaplan-Meier disease-free survival curves of CVC episodes showed no appreciable survival advantage by race at the healthcare system-wide level (Figure 1) or at the facility-specific level at three out of four hospitals (Supplemental Figure 1). At Hospital C, NH White Race appeared protective after about 17 days of CVC use, at which point most CVC episodes had been censored (Supplemental Figure 1). In an adjusted cox regression model accounting for TPN use and CVC-risk level, the risk of CLABSI in the first 14 days appeared slightly higher among NH Black patients when compared to NH White patients (aHR 1.19; 95% CI: 0.94, 1.51), while NH Black race exhibited a slightly protective effect after 14 days (aHR 0.80; 95% CI: 0.54, 1.17). Neither finding reached statistical significance.

The primary analysis evaluating the relationship between SVI and CLABSI risk was limited to the 44,646 (87%) CVC episodes successfully geocoded and mapped to a census tract. The odds of having a CLABSI among the most vulnerable subset (top quartile) compared to less vulnerable patients (other quartiles) was no different for each of the four SVI themes (Table 4). Additional analysis exploring SVI as continuous values did not identify any SVI themes statistically associated with CLABSI occurrence (data not shown).

**Table 4.** Association between social vulnerability index (individual themes and overall) and risk of CLABSI

SVI	With CLABSI (N = 392)	Without CLABSI (N = 44254)	Odds ratio (95% CI)^a^
*Socioeconomic*			
High (>0.75) vs Lower	98 (25.0%)	11174 (25.2%)	0.99 (0.78–1.24)
*Race and Ethnicity and Language*			
High (>0.75) vs Lower	101 (25.8%)	10764 (24.3%)	1.08 (0.86–1.35)
*Household Composition and Disability*			
High (>0.75) vs Lower	139 (35.5%)	17393 (39.3%)	0.85 (0.69–1.04)
*Housing and Transport*			
High (>0.75) vs Lower	91 (23.2%)	9176 (20.7%)	1.16 (0.91–1.46)
*Overall SVI*			
High (>0.75) vs Lower	95 (24.2%)	11121 (25.1%)	0.95 (0.75–1.20)

Note. CLABSI, central line-associated bloodstream infection; SVI, social vulnerability index; CI, confidence interval.

a Logistic regressions estimated the unadjusted risk of CLABSI

## Discussion

Although we demonstrate differences in types of CVCs used between NH Black patients and NH White patients, we did not identify a difference in NHSN-defined CLABSI risk between these patients when adjusting for CLABSI risk inherent in type and configuration of the CVCs in use. Additionally, while we hypothesized that social vulnerability might be a better predictor of HAI risk than race and ethnicity, we also found no association between SVI and CLABSI risk.

Our findings are similar to those of Bakullari et al, who did not demonstrate a difference in rates of 6 HAIs between Black patients and NH White patients in a national database.^3^ Jeon et al found a small but statistically significant increased risk of healthcare-associated bloodstream infection, but not healthcare-associated urinary tract infection or healthcare-associated pneumonia, in NH Black patients compared to NH White patients at a tertiary referral hospital in Manhattan. Notably the difference in healthcare-associated bloodstream infection between races disappeared after controlling for other factors, with admission through the emergency department, primary payer status and comorbidity having the biggest impact.^5^ In contrast, more recent work by Gettler et al at an academic medical center in North Carolina found higher rates of both CLABSI and catheter-associated urinary tract infection amongst NH Black patients when compared with NH White patients.^6^

There are multiple potential explanations for the conflicting findings in the literature. First, there are differences between studies in the sources of data (and thus definitions of HAIs) utilized. Gettler’s work and our study utilized data reported to NHSN using standardized and more reliable definitions than ICD-coded data utilized in other studies. A notable difference between our study and that of Gettler et al was our ability to control for other CLABSI risk factors.^6^ We consider this to be the main strength of our study, since previous work using the same cohort of patients as ours identified multiple independent risk factors for CLABSI, specifically comorbidities, concomitant CVCs, type of CVC, and indication for CVC.^8^ Secondly, our study involves a cohort of more than 30,000 patients admitted to both urban and suburban medical centers. In fact, the CLABSI-free facility-specific survival curves appear to behave differently after about 14 days, with NH Black race appearing to be protective at two of the hospitals (Hospitals A and D) and NH White race appearing protective at the other two hospitals, with Hospital C reaching significance. This suggests there may be facility-specific differences or differences in region and/or hospital type that influence the likelihood of uncovering racial disparities with respect to HAIs (Supplemental Figure 1), though these findings must be interpreted with caution as they are unadjusted, and the differences by race only appear after most CVC episodes have been censored.

This study has several limitations. First, the data utilized to categorize race and ethnicity was extracted from documentation in the electronic medical record (EMR) through facility-specific intake procedures. Prior research at other medical centers has shown discordance between race documented in EMR when compared to patient report.^12,13^ With more complete and/or accurate data on race and ethnicity, it is possible that our findings would have been different. Regardless, deficits in the validity and completeness of data on race are not unique to our study.^5,6^ While we assigned an SVI value to each patient encounter, this value originates at the census tract level and therefore may not accurately reflect the individual vulnerability of a given patient from that census tract. However, for most census tracts, this variability is slight compared to the variability within entire counties and so is a reasonable proxy to use for this analysis. Additionally, SVI eliminates the potential information bias inherent in race assigned through the EMR. Our study carries the inherent limitations of a retrospective analysis, including the possibility that there were factors not controlled for that could have influenced our findings. Although this study encompassed multiple hospitals caring for a large and diverse patient population, our findings may not be generalizable to other regions in the country or other types of health systems. Finally, our study did not assess risk for CLABSI after discharge from acute care, when differences in knowledge of or access to infection prevention tools may influence risk greater than in the inpatient setting.

Race is increasingly recognized as a social construct rather than a genetically determined fixed attribute. However, we know that bias still exists in health care based on other’s perception of race.^1^ While we did not find disparities in (in-hospital) CLABSI risk based on race, ethnicity, or SVI, additional investigation is warranted to understand the drivers of the conflicting findings in the literature. Future research should assess for variation in the presence or degree of such disparities based on region or other healthcare system attributes to better target future interventions. Given the relative rarity of HAIs as an outcome measure, it may be more revealing to look for disparities in the adherence to process measures intended to reduce the risk of CLABSI, such as those encompassed in CVC insertion and maintenance bundles. In addition, HAIs can occur in the post-discharge setting either at home or at post-acute care facilities not captured by CDC’s NHSN well. Expanding studies to evaluate the impact of race and ethnicity on HAI incidence in the post-acute care setting is needed. Finally, attention at a national level to address the gaps in reporting of race and ethnicity in EMR is a critical step to advancing our knowledge in this area. While the Association for Professionals in Infection Prevention and Epidemiology (APIC) health inequalities and disparities task force recommended making race a mandatory field in NHSN, we need to also outline best practices for accurately categorizing race and ethnicity in the EMR.^14^

## Supporting information

Gottlieb et al. supplementary material Gottlieb et al. supplementary material

## Supplementary material

To view supplementary material for this article, please visit https://doi.org/10.1017/ice.2024.133
