# Supplementary figures and images for: Assessing for race, ethnicity, and socioeconomic disparities in central line-associated bloodstream infection risk in a large academic health system
Source: Infect Control Hosp Epidemiol. 2024 Oct 14;45(12):1385–90. doi: 10.1017/ice.2024.133 (PMC11663462; doi:10.1017/ice.2024.133)

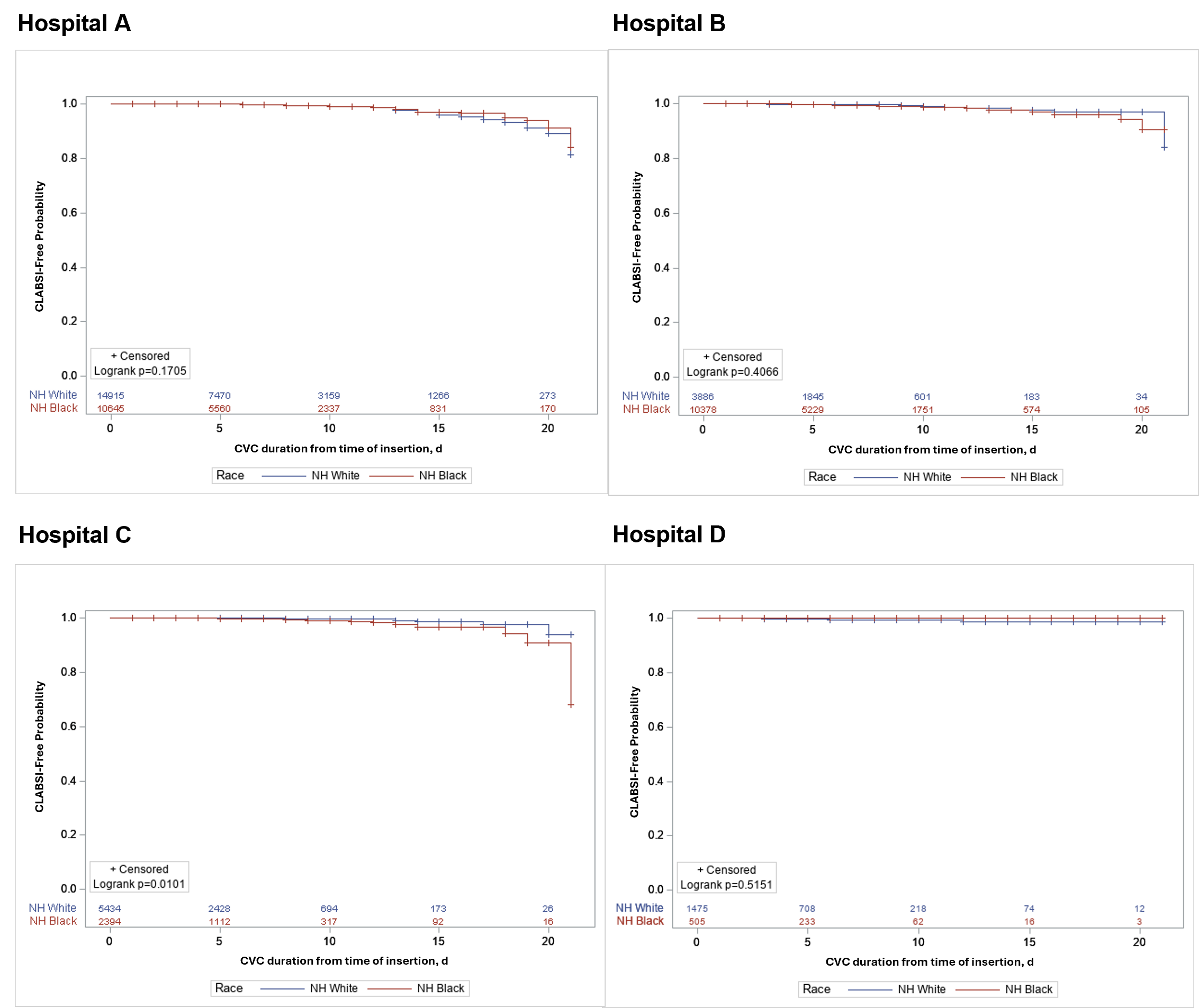

Supplement: Gottlieb et al. supplementary material [file S0899823X24001338sup001.tif]
